# Variation in MAPT is not a contributing factor to the incomplete penetrance in LHON

**DOI:** 10.1016/j.mito.2011.03.004

**Published:** 2011-07

**Authors:** Gavin Hudson, Patrick Yu-Wai-Man, Philip G. Griffiths, Rita Horvath, Valerio Carelli, Massimo Zeviani, Patrick F. Chinnery

**Affiliations:** a Institute for Human Genetics, Newcastle University, Newcastle upon Tyne, UK; b Department of Ophthalmology, Royal Victoria Infirmary, Newcastle upon Tyne, UK; c Department of Neurological Sciences, University of Bologna, Bologna, Italy; d Foundation Neurological Instituto C. Besta, Unit of Molecular Neurogenetics, Milan, Italy

**Keywords:** LHON, Mitochondria, Optic neuropathy, Tau, Neurodegeneration, MAPT

## Abstract

Leber's hereditary optic neuropathy (LHON) is a common cause of inherited blindness, primarily due to one of three mitochondrial DNA (mtDNA) mutations. LHON, which has an unexplained variable penetrance and pathology, is characterised by disruption of the mitochondrial respiratory chain ultimately resulting in degeneration of the retinal ganglion cells.

Phosphorylation of the tau protein is known to cause neurodegeneration and variation in *MAPT* has been associated with a range of neurodegenerative disorders. Given the relationship between *MAPT* variation and altered mitochondrial respiratory chain function, we hypothesised that *MAPT* variation could contribute to the risk of blindness in LHON mtDNA mutation carriers.

We studied *MAPT* variation in a large, well characterised LHON cohort, but were unable to find an association between *MAPT* genetic variation and visual failure in LHON mtDNA mutation carriers. Our findings suggest that genetic variation in *MAPT* is unlikely to make a major contribution to the risk of blindness among LHON mutation carriers.

## 1 Introduction

Leber hereditary optic neuropathy (LHON, OMIM#535000) is a common cause of inherited blindness that typically presents with bilateral, painless, sub-acute visual failure in young adult males (Carelli et al., 2004). The diagnosis is usually confirmed by molecular genetic analysis for one of three common mitochondrial DNA (mtDNA) mutations which all affect genes coding for complex I subunits of the respiratory chain: m.3460 G > A, m.11778 G > A and m14484T > C. However, not all patients harbouring a pathogenic LHON mtDNA mutation develop visual failure (Brown et al., 2001). Segregation analysis of LHON pedigrees implicates a two-locus model, with the mtDNA mutation as one locus and a modulating nuclear chromosomal locus (Bu and Rotter, 1991). However, attempts to identify a nuclear modifying gene by both genetic mapping and functional genomics have been inconclusive (Chen and Denton, 1991; Harding et al., 1995; Hudson et al., 2005).

Affected individuals develop focal degeneration of the optic nerve and present clinically with impaired color vision (dyschromatopsia), a dense visual field defect (central or caecocentral scotoma) and abnormal visual electrophysiology due to primary retinal ganglion cell loss (RGC) (Carelli et al., 2004). Although the primary biochemical defect involves complex I of the mitochondrial respiratory chain, the mechanisms leading to neurodegeneration remain unclear. Optical coherence tomography has shown an initial thickening of the retinal nerve fibre layer before the onset of visual symptoms, followed by progressive thinning corresponding to the axonal loss (Barboni et al., 2005). Recently, phosphorylated neuronal filament heavy chain (pNF-H), a prospective marker for neurodegeneration in Alzheimer's disease (Petzold, 2005; Petzold et al., 2008), was shown to be elevated in the serum of LHON patients (Guy et al., 2008).

Phosphorylation of the tau protein, encoded by the *MAPT* gene on chromosome 17, has been shown to result in neurodegeneration through the accumulation of neurofilamentray tangles (NFT). Variation in *MAPT* has been associated with a range of neurodegenerative disorders, providing evidence that disrupting tau homeostasis is sufficient to cause neuronal degeneration (Bugiani et al., 1999; Delisle et al., 1999; Rizzini et al., 2000), with particular variants tagging to two distinct disease related sub-haplotypes (H1 and H2) (Baker et al., 1999). Of particular interest, both *MAPT* variation and a biochemical defect of complex I also appear to be involved in the pathogenesis of Parkinson's disease.

Given the emerging evidence that tau-mediated neurodegeneration is caused by a combination of factors, including mitochondrial dysfunction (Thies and Mandelkow, 2007), it is plausible that *MAPT* variation contributes to the risk of blindness in LHON mtDNA mutation carriers.

## 2 Materials and methods

We studied 10 common *MAPT* (NM_001123066.3) single nucleotide polymorphisms (SNPs): (rs9486, rs242562, rs2435205, rs2435207, rs1467966, rs1800547, rs242557, rs2435211, rs2471738, rs3785883) in a European cohort consisting of 288 patients with LHON and 336 unaffected LHON family members (from 214 unrelated families) all harbouring a homoplasmic primary LHON mtDNA mutation. The clinical phenotype was determined by a local ophthalmologist, and all subjects were homoplasmic for one of the primary LHON mtDNA mutations (patients — m.3460G>A, n = 51, m.11778G>A, n = 201, m.14484T>C, n = 23, m.14495G>A, n = 1, and unaffected family members — m.3460G>A, n = 71, m.11778G>A, n = 226, m.14484T>C, n = 18, m.14495G>A, n = 0, 15257 = 21). Mutation status was confirmed by direct sequencing of the *MTND* genes or PCR-RFLP analysis as previously described (Taylor et al., 2001). Unaffected carriers were classified and included only if they had remained asymptomatic until > 30 years of age.

SNPs were selected using the following criteria: (1) substitutions predicted to affect *MAPT* function or shown to increase the risk of Parkinson's disease (Goris et al., 2007; Skipper et al., 2004; Tobin et al., 2008; Vandrovcova et al., 2009); (2) presence in control subjects at > 1.0% (dbSNP) (Sherry et al., 2001). *MAPT* H1 and H2 sub typing were performed by rs9486 allelic discrimination (Goris et al., 2007; Skipper et al., 2004).

The frequency of sequence variants was determined by primer extension of multiplex polymerase chain reaction products with the detection of the allele-specific extension products by matrix-associated laser desorption/ionization time of flight (MALDITOF; Sequenom, San Diego, CA) mass spectrometry.

Allele and genotype frequencies were compared using uncorrected Fishers exact test. Multiple logistic regression was performed to look for interactions between the variables. Age of onset was assessed as survivability using Kaplan–Meier, with comparisons made via Mantel–Cox log rank test. All analyses were carried out using SPSS software version 17.0 (SPSS Inc.). Statistical power was calculated using power for association with error (PAWE) (Gordon et al., 2003).

## 3 Results

### 3.1 MAPT haplotyping

The distribution of *MAPT* H1/H2 haplotypes and genotypes in both affected LHON cases and unaffected LHON mtDNA mutation carriers was not significantly different to published population controls (Skipper et al., 2004) (affected LHON cases versus published controls P = 0.613 and unaffected LHON mtDNA mutation carriers versus published controls P = 0.727, Table 1). There was no significant difference in the frequency of *MAPT* H1/H2 haplotypes (P = 0.656) or H1/H2 alleles (P = 0.638, Table 1) when affected LHON cases were compared to unaffected LHON mtDNA mutation carriers. Given the male predominance of affected LHON cases, we compared H- genotype and allele frequencies in males and females separately, but again were unable to find a significant association (Genotype; males P = 0.573 and females P = 0.626 alleles; males P = 0.455 and females P = 0.797).

### 3.2 Variant genotyping

There was no significant association between; rs242562, rs2435205, rs2435207, rs1467966, rs1800547, rs242557, rs2435211, rs2471738 or rs3785883 and visual failure in the European LHON mutation carriers when comparing alleles or genotypes (Table 2). Again, given the male bias in LHON, we compared the frequency of the *MAPT* variants in both males and females separately, but were unable to identify a significant association.

### 3.3 Effect of mitochondrial DNA background

The clinical penetrance of LHON is strongly associated with mtDNA haplogroup J (Hudson et al., 2007). With this is mind, we compared MAPT H-haplotypes and the common *MAPT* variants in both J and non-J haplogroups by logistic regression analysis, controlling for gender. This failed to identify an interacting association between background mitochondrial haplogroup J and H1, H2 and H1:H2 haplotypes and the presence of visual failure in LHON mtDNA mutation carriers (P value for H1 haplotype = 0.749, H2 haplotype = 0.190 and H1/H2 haplotypes = 0.553).

Similarly, we found no association between visual failure, the common *MAPT* variants and haplogroup J by logistic regression, again controlling for gender, (P = rs242562 = 0.749, rs2435205 = 0.357, rs2435207 = 0.731, rs1467966 = 0.760, rs1800547 = 0.594, rs242557 = 0.203, rs2435211 = 0.409, rs2471738 = 0.659 or rs3785883 = 0.665). There was no significant association identified with H-haplotypes (P value for H1 haplotype = 0.357, H2 haplotype = 0.151 and H1/H2 haplotypes = 0.909), or common MAPT variants when limiting to non-J haplogroups.

### 3.4 Age of onset

To assess the potential effect of H-haplotype and genotype on the age of onset of visual symptoms we compared H1 and H2 alleles to the age of onset of 250 symptomatic LHON patients (mean age of onset 22.60 years, StDev = 14.30). We were unable to identify a significant difference between H1 and H2 carriers (Mantel–Cox P = 0.623), further analysis showed no significant difference in age of onset when comparing *MAPT* common variants and age of onset (Mantel–Cox P = rs242562 = 0.762, rs2435205 = 0.621, rs2435207 = 0.346, rs1467966 = 0.289, rs1800547 = 0.416, rs242557 = 0.516, rs2435211 = 0.723, rs2471738 = 0.654 and rs3785883 = 0.251).

## 4 Discussion

We conducted a large case control study of *MAPT* variants previously shown to be associated with neurodegenerative disorders (Goris et al., 2007; Skipper et al., 2004; Tobin et al., 2008; Vandrovcova et al., 2009); based on the hypothesis that common variation in the *MAPT* gene is a major factor responsible for the incomplete penetrance in LHON. Previous studies have shown that the H1 haplotype is over represented in tauopathies (Goris et al., 2007; Skipper et al., 2004; Tobin et al., 2008; Vandrovcova et al., 2009), demonstrating increased risk to neurodegeneration, and the H2 haplotype has been shown to alter neuronal glucose utilisation (Laws et al., 2007), a factor known to be important in LHON (Ghelli et al., 2003; Hofhaus et al., 1996). However, we found no significant association to H-haplotypes and the presence or age of onset of the visual failure.

Previous studies have identified significant association to a number of *MAPT* SNPs, particularly rs1800547, rs2471738 and rs242557 (Tobin et al., 2008; Vandrovcova et al., 2009), to neurodegenerative disorders. We were unable to replicate these findings in LHON. Given an affected population size of 288 and a control population size of 336 our power to detect a > 10% difference in cases/control allele frequency was 0.942, and a 10% difference in genotype frequency was 0.893. With a 5% genotyping error (∑ 1 = Pr = 0.05), we would still be expected to detect a > 10% difference with an allelic power of 0.888 and a genotypic power of 0.817. We therefore conclude that it is highly unlikely that common *MAPT* variants play a major role in determining the variable clinical phenotype in LHON families. It is conceivable that *MAPT* variation plays a minor role, perhaps in combination with other genetic and environmental factors, but this will be extremely difficult to demonstrate given that LHON is a relatively rare disorder affecting ~ 1 in 14,000 males. Collecting a large enough cohort to show more subtle genetic effects would be a major challenge.

## Figures and Tables

**Table 1:** Comparison of H1/H2 haplogroups in LHON cases, LHON controls and published population controls (Skipper et al., 2004) (shown as frequency and percentage frequency in brackets, where A is symptomatic LHON mtDNA mutation carrier, U is asymptomatic LHON mtDNA mutation carrier and C* is published population controls. Probability is shown, P, by Fishers Exact test).

	Haplotype			
	H1	H2	H1/H2	P
A	178 (63.1)	19 (6.7)	85 (30.1)	0.656
U	197 (60.8)	28 (8.6)	99 (30.6)	
C*	262 (63.9)	16 (3.6)	143 (32.4)	
	H1	H2		
A	263 (71.7)	104 (28.3)		0.638
U	296 (80.7)	127 (34.6)		

**Table 2:** Allele (A) and genotype (G) comparison of common disease-associated *MAPT* variants in LHON mtDNA mutation carriers (where A is symptomatic LHON and U is asymptomatic mtDNA mutation carrier, P = uncorrected probability by Fishers Exact test).

		A1	A2	P	G1	G2	G3	P
rs242562		G	A		G	A	GA	
	A	201	182	0.621	76	57	125	0.522
	U	222	217		84	79	138	
rs2435205		A	G	0.498	A	G	AG	
	A	218	195		73	50	145	0.392
	U	232	229		72	69	160	
rs2435207		G	A	0.808	G	A	GA	
	A	247	99		173	25	74	0.777
	U	276	116		194	34	82	
rs1467966		T	C	1.000	T	C	TC	
	A	227	113		123	9	104	0.705
	U	268	135		148	15	120	
rs1800547		A	G	0.938	A	G	AG	
	A	224	113		120	9	104	0.799
	U	262	134		142	14	120	
rs242557		G	A	0.605	G	A	GA	
	A	215	132		102	19	113	0.584
	U	259	173		111	25	148	
rs2435211		T	C	1.000	T	C	TC	
	A	224	115		120	11	104	0.546
	U	224	115		153	17	113	
rs2471738		T	C	0.519	T	C	TC	
	A	249	73		179	3	70	0.698
	U	296	77		222	3	74	
rs3785883		G	A	0.919	G	A	GA	
	A	221	57		173	9	48	0.692
	U	255	66		196	7	59	
